# Opening up about Tissue Transglutaminase: When Conformation Matters More than Enzymatic Activity

**DOI:** 10.20900/mo.20180011

**Published:** 2018-11-22

**Authors:** William P. Katt, Marc A. Antonyak, Richard A. Cerione

**Affiliations:** 1 Department of Molecular Medicine, Cornell University, Ithaca, NY 14853, USA; 2 Department of Chemistry and Chemical Biology, Cornell University, Ithaca, NY 14853, USA

**Keywords:** tissue transglutaminase, GTP-binding, cancer, transamidation, cell death, conformational changes, protein crosslinking

## Abstract

Tissue transglutaminase (tTG), also referred to as type 2 transglutaminase or Gα_h_, can bind and hydrolyze GTP, as well as function as a protein crosslinking enzyme. tTG is widely expressed and can be detected both inside cells and in the extracellular space. In contrast to many enzymes, the active and inactive conformations of tTG are markedly different. The catalytically inactive form of tTG adopts a compact “closed-state” conformation, while the catalytically active form of the protein adopts an elongated “open-state” conformation. tTG has long been appreciated as an important player in numerous diseases, including celiac disease, neuronal degenerative diseases, and cancer, and its roles in these diseases often depend as much upon its conformation as its catalytic activity. While its ability to promote these diseases has been traditionally thought to be dependent on its protein crosslinking activity, more recent findings suggest that the conformational state tTG adopts is also important for mediating its effects. In particular, we and others have shown that the closed-state of tTG is important for promoting cell growth and survival, while maintaining tTG in the open-state is cytotoxic. In this review, we examine the two unique conformations of tTG and how they contribute to distinct biological processes. We will also describe how this information can be used to generate novel therapies to treat diseases, with a special focus on cancer.

## 1 INTRODUCTION

In 1957, Heinrich Waelsch discovered that liver extracts from guinea pigs would incorporate radio-labeled primary amines, such as cadaverine or lysine, into lysate proteins in a calcium dependent manner ^[1]^. It would eventually be determined that enzymatic reaction was mediated by the type 2 “tissue” transglutaminase (tTG or TG2, EC 2.3.2.13), a widely expressed protein which catalyzes transamidations between amides (generally from glutamine residues) and primary amines (often from lysine residues) ^[2]^. tTG has been extensively studied in the years since and found to be one of the major autoantigens in celiac disease ^[3–7]^, to be necessary for the survival and growth of cancer cells ^[8–13]^, and to be important for the formation of protein aggregates that are characteristic of Alzheimer’s and Parkinson’s diseases ^[14–18]^. The roles of tTG in various disease states have led to efforts to design small molecule inhibitors of the protein ^[19–22]^. These various studies have been extensively reviewed ^[2,23–29]^, and as such will be touched upon only briefly here. Instead, we will focus upon a comparatively less well examined and appreciated topic—how changes in the conformation of tTG impact its effects on cells.

**Fig. 1:**
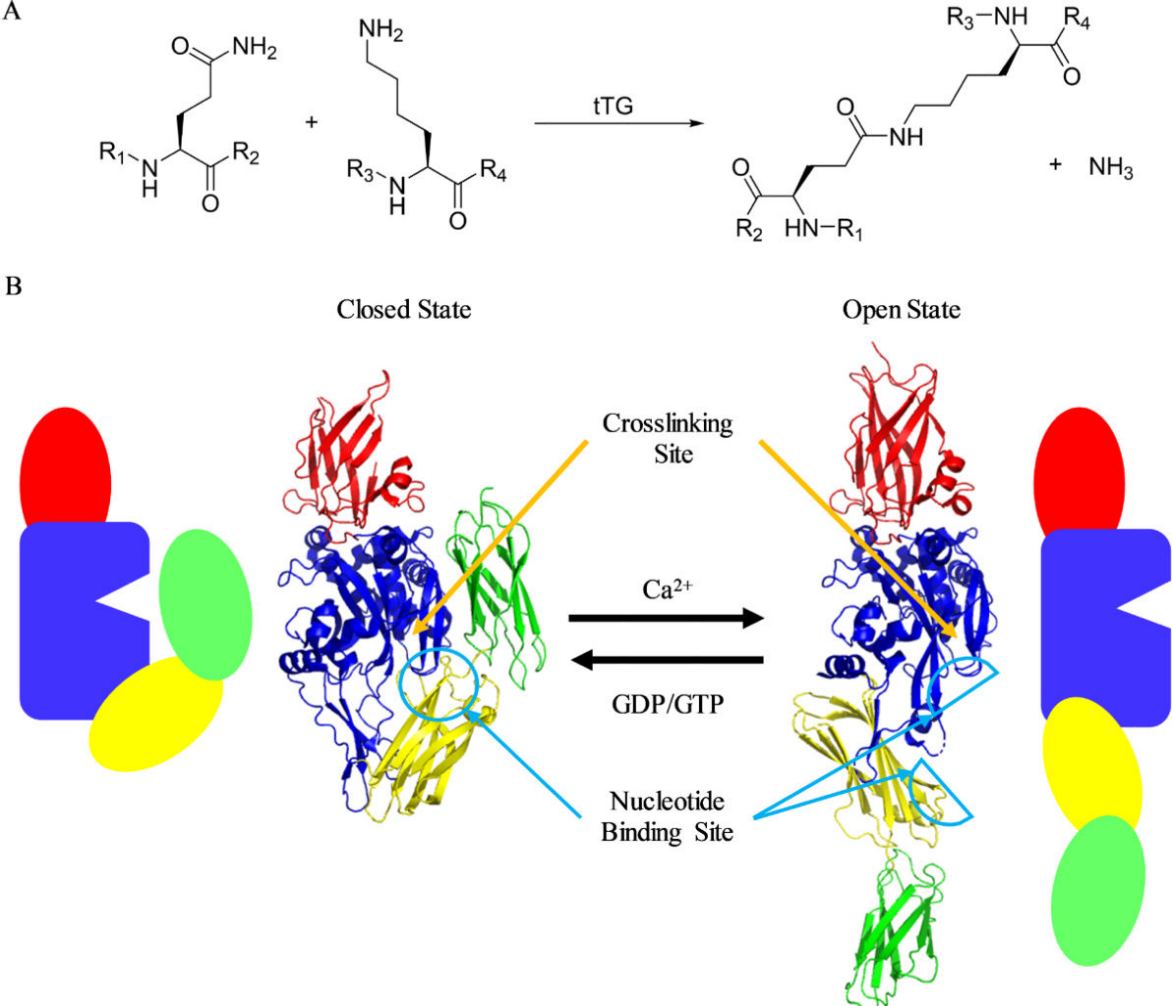
Structure and Function of tTG. (A) tTG catalyzes several different reactions: the most important of these is transamidation, or crosslinking of glutamine and lysine residues, which is shown schematically. (B) Crystal structures of tTG reveal two different conformational states. On the left, tTG is shown in the closed-state conformation (PDB code 231KV3). As the cartoon shows, the crosslinking substrate binding site (open wedge in blue crosslinking domain) is occluded. Addition of Ca2+ shifts the protein to the openstate conformation, shown on the right (PDB code 2Q3Z), which reveals this substrate binding site. An excess of GDP or GTP returns the protein to the closed-state. For either crystal structure, the crosslinking catalysis domain is indicated with an orange arrow, while the area where nucleotide would bind is circled and indicated with a blue arrow.

tTG has two distinct catalytic activities: a GTP-binding and hydrolysis activity, and a transamidation activity (Fig. 1A). Like many proteins, such as G-proteins or their coupled receptors, the function of tTG is dependent upon its conformation ^[30,31]^. However, unlike most proteins, whose conformation shifts quite subtly to facilitate signaling, tTG undergoes a significant structural shift which determines its activity. When bound to GTP or GDP, tTG adopts what is now referred to as the closed-state. Fig. 1B (left side) shows the crystal structure, and a cartoon representation, of the conformation that human tTG adopts when it is bound to a nucleotide, in this case GDP ^[32]^. This structure, the first solved for human tTG, revealed a compact protein comprising four domains: an N-terminal β-sandwich (red), a crosslinking-catalytic core (blue), and two C-terminal β-barrel domains (yellow and green). The structure clearly shows the binding site for nucleotides, and how substrate access to the crosslinking binding site, and key catalytic residue Cys-277, was occluded by the C-terminal β-barrels ^[33,34]^. However, upon binding calcium, tTG undergoes a dramatic conformational change. The nature of this change was demonstrated by Pinkas *et al.*, when they solved the crystal structure of tTG covalently bound to the gluten peptide mimetic inhibitor “Ac-P-DON-LPF-NH_2_” ^[35]^. This structure (Fig. 1B, right side) showed that the C-terminal β-barrels swiveled almost 180° from their start position, resulting in a nearly linear conformation for the protein, which allowed access of substrates to the crosslinking catalytic site. Interestingly, this conformational change eliminates the nucleotide binding site. The calcium-activated conformation of tTG is now commonly referred to as the “open-state”, while the nucleotide bound form of tTG is generally referred to as the “closed-state”.

GTP/GDP and Ca^2+^ are able to compete with one another to convert tTG from the closed- to open-state, and vice versa (Fig. 1B) ^[36]^. Given the relatively high levels of GDP and GTP in cells (approximately 500 μM ^[37]^), and relatively low concentrations of free Ca^2+^ (low nanomolar ^[38]^), it is generally assumed that intracellular tTG is predominantly in the closed-state, with a small portion being in the crosslinking-competent open-state ^[39,40]^. Conversely, extracellular Ca^2+^ is at a higher concentration than GDP or GTP, suggesting that extracellular tTG would adopt the open-state, and be crosslinking-competent. This view is somewhat complicated by the mildly oxidative conditions in the extracellular space, however. A triad of cysteine residues (Cys 230, Cys 370, and Cys 371) are able to make one of two disulfide bonds (C370–C230, or C370–C371) in oxidative conditions ^[41,42]^. Either disulfide bond reduces crosslinking catalytic activity, but oxidized tTG maintains a conformation similar to the open-state. However, it is currently unclear how the disulfide bonds, which stabilize the open-state conformation of tTG, block the catalytic activity of that conformational state.

Despite the two radically different conformations of tTG, most research has traditionally focused upon tTG’s catalytic protein crosslinking activity. tTG has typically been considered to be “active” or “inactive”, rather than “open” or “closed”. Given the many diverse proteins which serve as tTG crosslinking substrates (such as α-synuclein, enolase, myosin, RhoA, and synapsin 1 ^[43]^), concentrating upon crosslinking activity can provide significant insight. Indeed, for many of the diseases in which tTG plays a critical role (particularly neurological diseases and celiac disease), the description of protein crosslinking activity is entirely adequate. However, at least in the case of cancer, tTG’s conformation and activity must be considered individually.

## 2 SEVERAL ROLES OF TTG IN DISEASE

One of the earliest diseases in which tTG was found to play a role is Alzheimer’s disease. In the late 90’s, Johnson *et al.* found that tTG activity was increased in the brain of Alzheimer’s disease patients ^[18]^. Moreover, they found that its activity was increased specifically in the prefrontal cortex, where neurofibrillary tangles appear in Alzheimer’s patients, and not in the cerebellum, which is typically unaltered by the disease. They thus concluded that tTG might be at least partially responsible for the formation of these tangles. This conclusion has been generally supported in the years since. One of the most important initiating factors for Alzheimer’s disease is the formation of a soluble pool of the amyloid beta protein (Abeta), which is the primary component of the damaging neurofibrillary tangles, or plaques, that cause Alzheimer’s disease ^[44]^. While Abeta can self-assemble into such plaques, the necessary concentration for such assembly is much higher than physiological levels. However, tTG is capable of crosslinking Abeta into plaques at physiological concentrations ^[44]^, and colocalizes with Abeta in both human brain and the brain of mouse models of Alzheimer’s disease ^[45,46]^. Moreover, deamidation of Abeta by tTG increases its solubility, thus increasing its propensity for plaque formation ^[47]^. tTG also plays at least one indirect role, by crosslinking Apolipoprotein E (ApoE) ^[48]^. ApoE is able to protect against Abeta plaque formation by transporting Abeta out of the brain. However, crosslinking by tTG inactivates the protein, rendering it unable to clear Abeta.

Parkinson’s disease is a related disorder, and tTG plays similar, but unique, roles in this disease as well. Where Alzheimer’s disease is driven in part by a buildup of Abeta aggregates, Parkinson’s disease is caused by the aggregation of α-synuclein. In Parkinson’s disease, α-synuclein is incorrectly processed into β-pleated fibrils, which in turn aggregate to form cytoplasmic inclusions called Lewy Bodies ^[49,50]^. In 2003, it was found that tTG catalyzed the crosslinking of α-synuclein, both *in vitro* and in cell models ^[14]^. More recent studies have confirmed the importance of this interaction, showing that specific tTG inhibitors such as KCC009 can block α-synuclein aggregation in SH-SY5Y neuroblastoma cells ^[51]^, and that tTG and α-synuclein both localize to the endoplasmic reticulum in disease brain samples ^[49]^. Today, tTG is considered a prognostic marker for Parkinson’s disease ^[49]^. However, the role of tTG in Parkinson’s disease is somewhat debated. Segers-Nolton and colleagues demonstrated in 2009 that α-synuclein crosslinked by tTG formed aggregates substantially different from those found in typical Parkinson’s disease brain ^[50]^. The tTG mediated aggregates formed α-helices, rather than β-sheets like disease aggregates. Further, unlike disease aggregates, they were unable to disrupt phospholipid vesicles. This suggests that, in at least some situations, tTG may play a protective role in Parkinson’s disease, by consuming α-synuclein and forming non-toxic aggregates, rather than allowing it to form normal, disease causing Lewy Bodies. The ability for tTG to situationally promote survival or cell death will be revisited below, particularly in the context of cancer.

Almost simultaneously with the discovery of tTG as a participant in Alzheimer’s disease, it was reported that tTG was the major autoantigen in celiac disease ^[4]^. Celiac disease is an auto-immune disorder in which T-cells attack and damage the small intestine. This process is driven by gliadin, a protein in most grains, which precipitates an immune response. In 1997, Dieterich and coworkers found that in celiac disease, the T-cells respond primarily to antibodies for tTG ^[4]^. They further found that gliadin was a substrate for tTG, and that tTG was crosslinked to gliadin. They proposed that tTG crosslinking of gliadin formed antigenic complexes, and that these complexes are what the immune system responded to, a hypothesis that was confirmed one year later, and further characterized in the years since ^[52–54]^. Related research into tTG’s role in celiac disease has been quite active as well. Many studies have focused upon tTG antibodies, and crosslinked gliadin, as diagnostic tools ^[55,56]^. Others have investigated systems in which activated tTG might be inhibited to model celiac disease, with one example being Caco-2 intestinal cancer cells, which express crosslinking-competent tTG on their surface ^[57,58]^. One particularly interesting study in 2016 showed that thioredoxin-1 is released by macrophages exposed to inflammatory stimuli in sufficient quantity to reduce the tTG C370–C371 disulfide bond, activating the enzyme ^[59]^. Since inflammatory conditions are present in celiac disease gut, this effect essentially creates a self-stimulating loop in which activated tTG leads to inflammation, which then activates more tTG. Celiac disease has historically been one of the most important areas of tTG research and indeed, the only inhibitor of tTG currently in clinical trials targets celiac disease ^[28]^.

In the diseases discussed so far, the role of tTG is comparatively straightforward. Its predominant function is to crosslink a specific protein in a detrimental way. Open-state, crosslinking competent tTG helps promote the disease, while closed-state tTG would be expected to have no effect. Its roles in cancer are far more diverse. For example, tTG has been shown to play roles in cancer cell adhesion, migration, and invasion via its interactions with fibronectin. tTG binds to fibronectin, and crosslinks it to various surfaces, allowing cells to adhere ^[60–62]^. Matrix metalloproteinase can then break these crosslinks, and in combination with tTG crosslinking this allows for cell motility ^[63]^. Similarly, tTG is thought to play a role in vesicle trafficking by helping to dock extracellular vesicles (microvesicles) generated by aggressive cancer cells to fibroblasts, through its ability to bind and crosslink fibronectin on the vesicle surface ^[9]^. This docking event can then be blocked by inhibiting its crosslinking activity. However, by far one of tTG’s most fascinating and complex roles is in cell survival.

Proteins involved in cell survival tend to promote either survival or cell death, but not both. However, a small number of proteins are capable of triggering both pathways. Proteins such as the cyclins, CDK1 and CDK2, the Bcl-2 family, and the Myc family have all been found to promote both apoptosis and cell proliferation under assorted conditions ^[64]^. Like these proteins, tTG can promote either cell survival or apoptosis, depending upon the physiological context ^[2,25]^. As a pro-survival protein, the crosslinking-competent, open-state form of tTG has been shown to crosslink pRB (a pro-apoptotic protein), causing it to oligomerize and thus lose its activity ^[65,66]^. This is analogous to its role in Alzheimer’s disease, crosslinking ApoE ^[48]^. However, closed-state tTG is able to sequester c-Cbl, and block ubiquitinylation and subsequent degradation of the EGF receptor, thereby also promoting cell growth and survival ^[67]^. Thus, both open- and closed-state can tTG promote survival depending upon the specific conditions. The same is true of its pro-apoptotic functions. In pancreatic cancer cells treated with the calcium ionophore A23187, tTG was shown to adopt the crosslinking-active open-state and to then facilitate release of the apoptosis-inducing factor from mitochondria, promoting cell death ^[68]^. In contrast, ectopically expressed tTG in SH-SY5Y cells, which presumably exists in the closed-state, was found to promote apoptosis following osmotic shock or staurosporine treatment ^[69]^. Perhaps the most exciting of these survival-related roles, however, is the inherent cytotoxicity of the open-state of tTG.

## 3 CYTOTOXIC OPEN-STATE OF TTG

**Fig. 2:**
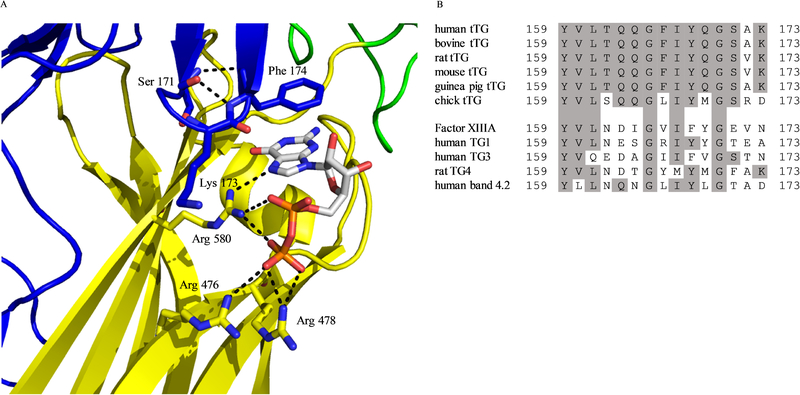
The nucleotide binding pocket of tTG. (A) Key residues in the nucleotide binding site of tTG (PDB code 1KV3). Arg 580 hydrogen bonds to several parts of the bound GDP nucleotide, while Phe 174 makes a π-stacking interaction with the guanine ring system. Ser 171 does not interact directly with the bound GDP, but forms hydrogen bonds to Phe 174 which may be important to stabilize its π-stacking interaction with the nucleotide. (B) Alignment of the nucleotide binding residues from the crosslinking domain of tTG across different species, and between different TG family members. T162, Q163, Q164, F166, Q169, and K173 are all highly conserved among tTG variants, but poorly conserved among TG family members. Figure panel adapted from ^[71]^.

The idea that maintaining tTG in the open-state could be detrimental to cells began with two studies published in the late 2000s. One report by Datta *et al.* showed that ectopically expressing mutant forms of tTG deficient in GTP-binding ability resulted in cell death ^[70]^. Specifically, recombinant forms of tTG with mutations in residues Arg 476, Arg 580, and Lys 173, were all shown to have diminished GTP binding compared to wild-type tTG (Fig. 2A). The greatest decreases in nucleotide binding were observed for the R580L and R580K mutants, and when transfected into NIH 3T3 fibroblasts or HeLa cervical carcinoma cells, nearly half of the cells died within 24 h. In contrast, introduction of wild-type tTG into those same cells did not induce cell death. It was further shown that the cell death caused by mutant forms of tTG that lack GTP-binding occurred independently of their crosslinking activity, as the expression of forms of tTG that were deficient in GTP-binding and protein crosslinking activity (e.g., the tTG R580L C277A double mutant) still induced cell death. They concluded their study by showing that the cell death was caspase-independent, and as such was presumably not apoptosis.

The second study did not examine tTG related cytotoxicity, but instead determined the conformation that different tTG mutants adopted. The key finding was that mutating Arg 579 in rat tTG (homologous to Arg 580 in human tTG) to alanine caused the enzyme to adopt the open-state ^[40,72]^. Thus, it was likely that the mutants used by Datta were also in the open-state. Combining the findings from these two reports suggested that the open-state conformation of tTG might be sufficient to elicit cytotoxicity.

Gozde Colak *et al.* put this theory to the test in 2011, when they studied the ability of various mutant forms of tTG to interfere with oxygen-glucose deprivation-induced cell death in immortalized mouse striatal cells ^[39]^. The authors generated cells that stably expressed tTG wild-type, tTG C277S (crosslinking deficient), or tTG R580A (GTP-binding deficient). Although the ectopic expression of tTG R580A in striatal cells was not sufficient to induce cell death as it did in HeLa or NIH 3T3 cells ^[70]^, it was shown that these cells were much more susceptible to glucose-oxygen deprivation mediated cell death compared to cells expressing tTG wild-type or the tTG C277S mutant. The authors then treated each of the cell lines that they had generated with two transamidation inhibitors: Cp4d, a reversible small molecule which has little effect on tTG conformation ^[73]^, and NC9, a bulkier, irreversible peptidomimetic compound which they presumed would stabilize tTG in the open-state. Cp4d treatment had little effect on the sensitivity of the assorted cells to glucose-oxygen deprivation-induced cell death. However, NC9 caused the tTG wild-type and tTG C277S expressing cells to undergo a greater degree of cell death under the same conditions, providing further support to the idea that the conformation of tTG was responsible for the cell death enhancement.

However, despite the importance of tTG in various diseases, and the puzzle posed by a pro-survival protein inducing cell death when held in its open-state, there is little mechanistic understanding regarding how open-state tTG promotes cytotoxicity. It may be that the open-state form of tTG either interacts with a key binding partner to induce cell death, or is no longer able to associate with a closed-state binding partner which would prevent cell death. Indeed, a number of findings from the literature may provide clues as to how open-state tTG induces cytotoxicity. We will therefore begin by examining the key assays used to assess tTG conformation, and several important tTG mutants which have been used to query the biological properties of the open- and closed-states. We will then discuss some potential binding partners of tTG, including several which bind preferentially to one conformational state or the other, as well as examine some roles generally ascribed to closed-state tTG but which might in fact be due to open-state tTG. Finally, we will discuss several small molecules which have been described in the literature and shown to stabilize the open-state conformation of tTG.

## 4 IDENTIFICATION OF TTG MUTANTS WHICH ADOPT EITHER THE OPEN-OR CLOSED-STATE

Many studies examining the open-state conformation of tTG have focused on guanine nucleotide-binding deficient Arg 580 mutants of tTG. However, several other tTG mutants have been discovered which stabilize the open-state, whereas other tTG mutants have been found to promote the closed-state conformation. In 2006, Begg and colleagues demonstrated a number of methods to specifically study tTG conformation ^[40,72]^. One of the most important of these is to monitor its guanine nucleotide binding capability. The nucleotide binding site of tTG is only fully accessible in the closed-state conformation. Begg and coworkers monitored nucleotide binding by two mechanisms. The first involved incubation of tTG with radioactive [α−^32^P] GTP, followed by SDS-PAGE and autoradiography.

The second mechanism involved isothermal titration calorimetry (ITC), in which small aliquots of GTP-γS were added to tTG, and the heat evolved during binding was measured at each step to determine the binding constant for the ligand and the molar stoichiometry for the GTP-γS-tTG interaction. Other laboratories have since altered this technique to monitor the fluorescence of bodipy-GTP-γS ^[67,70]^. The bodipy fluorophore is environmentally sensitive, and has a much higher emission in hydrophobic environments (such as the nucleotide binding pocket of tTG) compared to water. Further, by making use of a fluorophore, the assay becomes more amenable to high-throughput screening than those involving radioisotopes or ITC ^[70]^.

A second major approach involved monitoring the proteolytic degradation of tTG. In 1987, Achyuthan and Greenberg demonstrated for the first time that GTP was able to inhibit tTG crosslinking activity. Moreover, they showed that GTP protected tTG from proteolytic degradation by trypsin, and that addition of CaCl_2_ reversed this effect ^[36]^. These findings can now be explained as follows: CaCl_2_ enhances the formation of the open-state of tTG, and thus exposes numerous peptide bonds which are sensitive to trypsin. Guanine nucleotide binding to tTG has the opposite effect, causing tTG to adopt the closed-state, thus making its proteolytic sites less accessible. Begg and coworkers then showed that the R579A mutant of tTG was far more susceptible to proteolysis by trypsin or by calpain ^[40]^. Begg and colleagues went on to use a third assay technique, monitoring the electrophoretic shifts of tTG, given that tTG migrates further via native-PAGE when bound to nucleotide than when in the nucleotide free state, consistent with a more compact protein (the closed-state) migrating more rapidly than a less compact protein (the open-state).

The GTP-binding and proteolytic degradation assays have been important in assaying tTG mutants at positions other than Arg 579/580 in recent years. In 2006, Begg and colleagues examined several different mutants, focusing upon residues in the nucleotide binding site identified by Liu *et al.* ^[32,40]^. They studied residues thought to bind nucleotide, and nearby residues which would not be expected to directly associate with the ligand. As Table 1 shows, in addition to the Arg 579 mutant, they found the F174A mutant to be deficient in nucleotide binding, and to be digested by trypsin in the presence of GTP-γS. Phe 174 appeared to be involved in a pi-stacking interaction (Fig. 2A), and this was verified by examining tTG F174W, which resisted proteolysis and was able to bind nucleotide. The R478A mutant was found to have partially reduced nucleotide binding, while tTG R476A and tTG S171A bound nucleotide as well as the wild type protein. As shown in Fig. 2A, Arg 580 in wild type tTG makes several hydrogen bonds to GDP, while Arg 478 binds only to the terminal phosphate. Arg 476 binds more poorly to the terminal phosphate of GDP, and Ser 171 makes no hydrogen bonds to the GDP molecule. These results were therefore consistent with the crystallographic data.

Datta and colleagues studied several similar mutants when they demonstrated the cytotoxicity of tTG R580K ^[70]^. In particular, they found that tTG R478L almost completely lost the ability to bind nucleotide (bodipy-GTP-γS), suggesting that the steric bulk of leucine was responsible for the impaired binding. They also found that tTG R478L was cytotoxic upon ectopic expression in NIH 3T3 or HeLa cells, similar to tTG R580L and tTG R580K.

An earlier study by Iismaa *et al.* demonstrates that it is important to consider multiple single-point mutations when analyzing the importance of a particular residue to tTG nucleotide binding and/or conformation ^[71]^. The authors attempted to identify residues involved in nucleotide binding based on species homology (Fig. 2B). They examined Ser 171 and made two different mutations: S171E and S171C. Interestingly, while S171C was without effect, the S171E substitution completely prevented nucleotide binding ^[40]^. Despite the conservation of Gln at three different positions in the examined region (Fig. 2B), neither tTG Q169L, tTG Q164L, tTG Q163L, or tTG Q163D showed any loss of nucleotide binding ability when assayed with [α−^32^P] GTP, and these mutants exhibited only a moderate loss of binding ability when assayed with [^35^S]GTP-γS, suggesting the residues were only minimally involved with GTP binding. Liu’s crystal structure would later verify that these residues were not involved in nucleotide binding (Fig. 2A) ^[32]^.

**Fig. 3:**
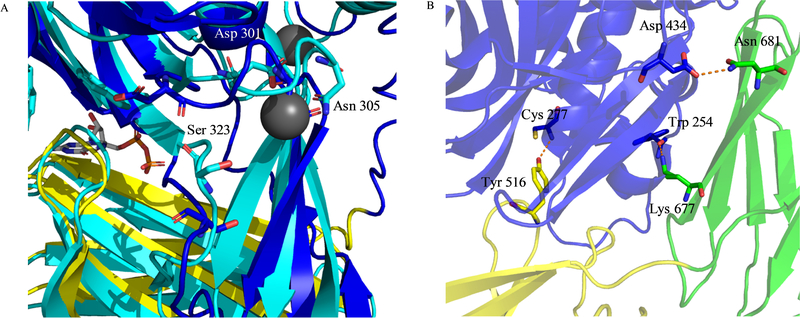
Important bonds which stabilize the conformations of tTG. (A) One of three calcium binding sites identified for TG3. In TG3 (cyan, PDB code 1NUD), calcium is bound by Asp 301 and Asn 305. Upon binding these residues, it pulls on Ser 323 to shift the nearby loop and provide access to the substrate binding site. In blue, tTG (PDB code 1KV3) is overlayed. tTG has Asp and Asn residues very close to those in TG3. (B) Key hydrogen bonds which stabilize the closed-state of tTG. Tyr 516 hydrogen bonds to Cys 277 (left), while on the other face of the protein, Asp 434 and Asn 681 form one hydrogen bond, and Trp 254 and Lys 677 form another. Of these latter four bonds, only Trp 254 makes the bond via backbone atoms.

Other studies have focused on residues outside the nucleotide binding site of tTG, to further demonstrate the intricate mechanisms which control tTG conformation. Zhang *et al.* examined the tTG double-mutant D306N/N310A ^[67]^. These residues lie in “site II”, one of three calcium binding sites thought to exist on tTG. In 2002 and 2003, Ahvazi and coworkers reported a series of crystal structures of the highly homologous TG3, which revealed binding sites for three calcium ions (Fig. 3A) ^[75,76]^. Datta *et al.* in turn showed that mutations of homologous residues on tTG at two of these binding sites resulted in a significant reduction in calcium-stimulated crosslinking activity, with the D306N/N310A double mutant completely abolishing catalytic activity ^[8]^. Zhang showed that purified tTG D306N/N310A adopted a conformation similar to that of wild type tTG, based on their mutual ability to bind bodipy-GTP-γS and to resist proteolysis by trypsin. One further mutant was studied by Zhang, tTG C277V. While a 2011 study suggested that tTG C277S was largely identical to wild-type tTG in terms of induction of cell death ^[39]^, Zhang showed that tTG C277V was susceptible to digestion by trypsin, and significantly impaired in nucleotide binding (albeit not to the same extent as tTG R580K). Begg had also shown that tTG C277A was unable to bind guanine nucleotides, and claimed the same was true for tTG C277S ^[40]^. This raises questions as to whether the C277V, C277A, and C277S mutations have different effects upon tTG conformation, whether a partial decrease in nucleotide binding capacity is sufficient to stabilize the tTG open-state in cells, and whether or not all open-state tTG mutants are cytotoxic. These questions will hopefully be addressed in future studies.

Begg *et al.* also studied a tTG point mutant not directly related to nucleotide binding ^[40]^. The authors had identified Tyr 516 as making a hydrogen bond with Cys 277 in closed-state tTG (Fig. 3B), and postulated that it was partially responsible for blocking access of crosslinking substrates to Cys 277. In agreement with others, the authors found that Cys 277 mutants were less capable of binding guanine nucleotide, as monitored in this case by electrophoretic shift on native-PAGE gels. Similar results were obtained for the mutants tTG Y516C and tTG Y516F. Combined with the results from Zhang, which showed that tTG C277V had a reduced nucleotide binding capacity ^[67]^, this suggests that interference with the Cys 277/Tyr 516 hydrogen bond may result in a tTG conformation that is intermediate between the open- and closed-states.

In 2016, Singh *et al.* described small-angle X-Ray scattering (SAXS) studies on tTG and its R580K mutant ^[74]^. In these studies, they were clearly able to demonstrate that wild-type tTG in solution adopts a closed-state conformation, similar to the crystalized form identified by Liu ^[32]^, while the tTG R580K mutant in solution adopted an open-state conformation, similar to that described by Pinkas ^[35]^. This not only further verified that tTG Arg 580 mutants adopt an open-state conformation, as suggested by biochemical analyses ^[40,72]^, but also demonstrated that the marked conformational changes occurring within tTG were not simply due to crystallization artefacts. Singh *et al.* also designed new tTG mutants which stably adopted the open-state. Rather than targeting GTP-binding residues, as in previous studies, they targeted key hydrogen bonds between the catalytic core domain and the C-terminal β-barrel (Fig. 3B). Two hydrogen bond pairs were targeted: one between Asp 434 and Asn 681, and a second between Trp 254 and Lys 677. Thus, four single-point mutants were prepared: tTG D434A, tTG N681A, tTG K677A, and tTG W254A. While all could be transiently expressed in NIH 3T3 cells, only tTG W254A and tTG N681A could be generated as recombinant proteins. These latter two mutants were unable to bind bodipy-GTP-γS, strongly suggesting that they adopt the open-state. This was supported by their high sensitivity to degradation by trypsin. Although useful SAXS data could not be obtained for tTG N681A, the SAXS profile for tTG W254A suggested a dimer of tTG molecules in the open-state conformation. Each of the four tTG single-point mutants was cytotoxic when expressed in NIH 3T3 cells.

While Begg’s work helped to establish the basic assays most commonly used to assess tTG conformation ^[40,72]^, and Singh demonstrated that these assays were truly reflecting the solution state of tTG ^[74]^, neither study directly addressed the conformation of tTG in cells. Caron and coworkers helped to address this issue in 2012, when they reported a tTG-based biosensor, tagged at the N-terminus with mCerulean, (mCer) and at the C-terminus with yellow fluorescent protein (YFP). This mCer-tTG-YFP was monitored by FRET, and the authors were able to observe a reduction in signal (*i.e.*, upon tTG adopting the open-state) when the protein was exposed to Ca^2+^, by treating the cells with drugs such as NC9 that stabilize the open-state conformation of tTG, or by testing the mCer-tTG R580A-YFP mutant ^[73]^. Taken together, these studies strongly suggest that tTG adopts two markedly different conformations, depending upon the presence of calcium or guanine nucleotide, and that these conformations have very different cellular effects.

These studies have further laid the groundwork for studying tTG conformation by describing the methods by which tTG conformation can be monitored, by highlighting a number of sites which can be mutated to promote the open-state of tTG, and by demonstrating with several different tTG mutants that the open-state of the enzyme is cytotoxic when constitutively maintained. None of these studies answered the question of how cytotoxicity arises upon the expression of open-state tTG. However, some pertinent clues are available in the literature, as described below.

## 5 BINDING PARTNERS OF TTG DEPEND UPON ITS CONFORMATION

Upon finding that the open-state tTG induces cell death, the initial assumption was that these effects were due to unregulated crosslinking, and indeed, this was one of the earliest hypotheses tested. Cys 277 is an essential residue for tTG-catalyzed crosslinking activity, and mutation of this residue results in a crosslinking-defective protein ^[2]^. However, it was found that Cys 277 mutants had little or no effect on cell survival, while Arg 580 mutants decreased cell survival ^[39,70]^. Moreover, Datta *et al.* showed that the tTG double-point mutants R580L/C277A and R580K/C277A were able to induce NIH 3T3 or HeLa cells to undergo cell death as effectively as the Arg 580 single-point mutant ^[70]^. Colak *et al.* demonstrated that causing tTG R580A to localize to the nucleus by attaching a nuclear localization sequence eliminated its cytotoxic potential, at least as monitored by the release of LDH in glucose-oxygen deprived cells, and showed that NC9, an irreversible peptidomimetic inhibitor of tTG, caused both wild type tTG and tTG C277S to enhance LDH release, like the case for the tTG R580A mutant. Collectively, these findings strongly suggest that crosslinking activity is not necessary for the cytotoxic effects of open-state tTG, and that the toxic effects must then arise through another mechanism.

Given the above results, another possibility for explaining the cytotoxicity of open-state tTG is that it binds to a protein in the cytosol that helps to initiate a cell death pathway. However, the transdab database lists dozens of potential substrates and interaction partners for tTG ^[43]^. Further, the literature contains no direct evidence of open-state tTG binding to a specific effector which then promotes cell death. A different possibility, however, is that closed-state tTG binds to a protein in a way that promotes cell survival, and open-state tTG is somehow incapable of undergoing this interaction.

In 2013, Zhang *et al.* demonstrated that c-Cbl, an E3 ubiquitin ligase, bound selectively to tTG in the closed-state. Immunoprecipitation assays showed that c-Cbl would bind to wild-type tTG or to the “site II” mutant D306N/N310A, but not to either tTG R580K or tTG C277V ^[67]^. As previously discussed, the latter two mutants adopt predominantly open-state conformations, while the former mutant adopt predominantly closed-state conformations (Table 1). It was further demonstrated that two tTG inhibitors, the irreversible peptidomimetic Z-Don and the alternate substrate MDC, decreased EGFR expression, and increased EGFR ubiquitination, when applied to U87 MG or LN229 brain cancer cells, and that knockdown of tTG had similar effects. Finally, it was shown that MDC could directly block the interaction of tTG and c-Cbl as monitored by immunoprecipitation, and MDC stabilized the open-state in a dose-dependent manner. These findings showed that the closed-state of tTG was specifically responsible for promoting cell survival by sequestering c-Cbl and preventing EGFR degradation.

While the open-state tTG is incapable of binding c-Cbl, and thus promoting cell growth and survival, this lack of binding alone would not promote cell death. However, the possibility exists that open-state tTG might be able to dimerize with other tTG molecules, and cause those to adopt the open-state as well. Support for such a mechanism comes from a recent study by Kim *et al.*, in which the authors demonstrated that tTG forms a stable dimer in its open-state at temperatures above those used for crystallization studies (e.g., 30 °C) ^[77]^. The authors began by demonstrating that recombinantly expressed tTG formed dimers, and higher order polymeric structures, in a temperature-dependent manner, as read out by native PAGE. They then presented a SAXS profile matching the open-state tTG for both the monomeric and dimeric forms of the protein, and then used mass-spectrometry analysis of trypsin-digested monomeric or dimeric tTG to identify residues Ile 593–Lys 600 as the dimerization domain of tTG.

While this study would suggest that open-state tTG might effectively sequester wild-type tTG in cells, and thus prevent access by endogenous tTG to binding partners such as c-Cbl, there are a number of provisos which must be considered. These experiments were conducted in the presence of just 5 μM GTP ^[77]^, even though cells have almost 100X that concentration of GTP ^[37]^, and so there was a greater opportunity for the tTG to adopt the open-state than one might expect in cells. Further, these results are in conflict with experiments showing that mCer-tTG-YFP exhibited a strong FRET signal in cells, while mCer-tTG R580A-YFP did not, strongly suggesting that the former protein construct adopted the closed-state ^[73]^. Of course, it is possible that the mCer and YFP adducts altered the normal conformational equilibrium of tTG, however, another possibility is that the differences in readout are due to fundamental differences between recombinantly expressed tTG and tTG in the cellular environment. Also, the residues identified as the binding domain for tTG dimerization (593–600) are exposed on the surface in both the open-state and closed-state tTG ^[32,35]^. Thus, one would expect that dimerization could occur in either conformation, or even that hetero-dimerization of open-state and closed-state tTG might occur along that surface. As such, while reduction in binding between the closed-state tTG and its partners may be an important factor in the ability of open-state tTG to induce cytotoxicity, it seems unlikely to be the sole determinant in all situations. Further, to our knowledge, no experiment has yet been conducted to determine if constitutively open-state tTG is able to sequester wild-type tTG in cells, and thus prevent its interaction with effectors such as c-Cbl.

## 6 TTG AS THE G-PROTEIN Gα_H_

The work by Zhang *et al.* provided one example of how a specific conformation of tTG (the closed-state) was necessary to bind to a specific target (c-Cbl). However, other examples may be related to reports that tTG functions as a G-protein ^[78]^. As discussed above, in the closed-state, tTG binds to GDP or GTP, and is able to hydrolyze GTP to GDP, similar to classical G-proteins. Indeed, closed-state tTG was initially referred to as Gα_h_. In their study demonstrating tTG and Gα_h_ to be the same protein, Nakaoka and colleagues also showed that tTG bound to the α1B-adrenergic receptor, a G-Protein coupled receptor (GPCR), and was involved in receptor-mediated signaling events ^[78]^. The authors observed enhanced receptor signaling when both the receptor and tTG were transfected into COS-1 cells, and that the receptor could be co-immunoprecipitated with tTG from cell lysates. Nakaoka also demonstrated that addition of GTP-γS to the lysates caused much less α1B-adrenergic receptor to bind to tTG. Baek *et al.* reported similar findings when they demonstrated a binding relationship between tTG (Gα_h_) and another GPCR, the oxytocin receptor ^[79]^. tTG was able to bind to the receptor, as measured by immunoprecipitation experiments, but significantly less binding was measured in the presence of GTP-γS.

**Fig. 4:**
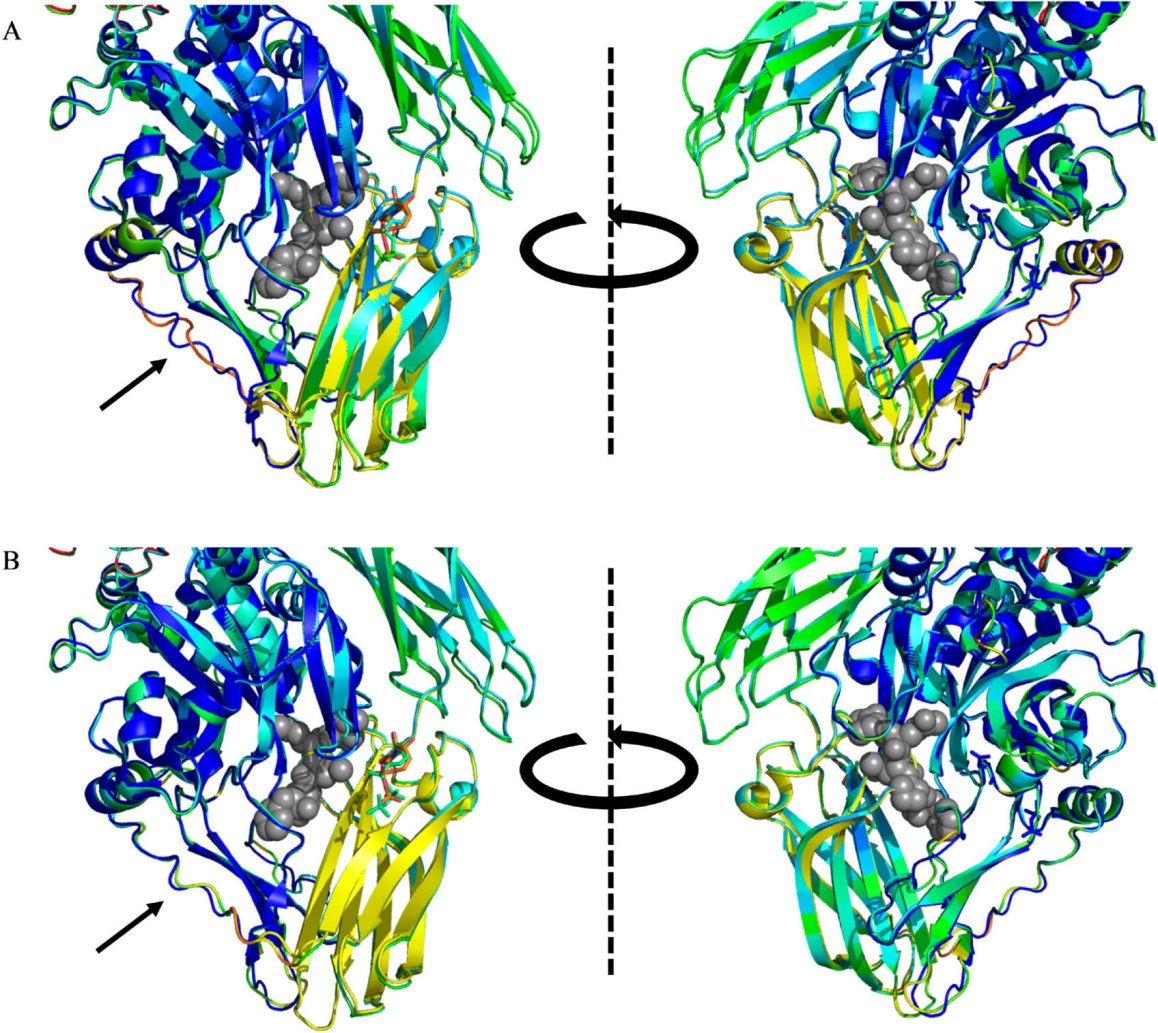
The structure of tTG does not change upon binding GDP or GTP. (A) Overlayed crystal structures of GDP-bound tTG (PDB code 1KV3, blue, yellow, and green) and GTP-bound tTG (PDB code 4PYG, colored by temperature factor, redder shades suggest more uncertainty in residue position). Gray spheres show the position of an open-state bound peptide sequence overlayed from open-state tTG (PDB code 2Q3Z). There is almost no change in the structure of tTG when GTP is bound rather than GDP. The only visibly changed loop (black arrow) also has very high temperature factors in the GTP-bound structure. (B) Overlayed crystal structures of GDP-bound tTG [same as in (A)] and ATP-bound tTG (PDB code 3LY6, colored by temperature factor). The same trends are present as in (A), save that the minimally varied loop (black arrow) has lower temperature in the ATP-bound structure, and perfectly overlays with the GDPbound structure.

Studies such as those by Nakaoka and Baek are consistent with tTG acting as a classical G-protein, where the GDP-bound form would bind to the receptor/ exchange factor, and the GTP-bound form would dissociate in order to engage downstream effector proteins. However, unlike the case for G-proteins, no structural differences have been observed between GDP- and GTP-bound tTG to explain why it would dissociate from a receptor upon nucleotide exchange (Fig. 4A) ^[32,80]^. Similarly, tTG has been shown to bind ATP, though this again causes no notable structural changes to the protein (Fig. 4B) ^[81]^. Given that tTG does not have loops analogous to switch-regions in classical G-proteins, it is uncertain how the binding of different nucleotides could change the binding affinity between tTG and assorted effectors.

Interestingly, in 2001, Park and coworkers demonstrated that tTG isolated from the cytosolic or membrane fractions of mouse heart cells had distinctly different properties, with cytosolic tTG having better crosslinking activity than membrane tTG, while membrane associated tTG exhibited higher GTP-ase activity than cytosolic tTG ^[82]^. These findings suggest some functions of tTG may be compartment-dependent, or that tTG undergoes some type of post-translational modification. Thus far, it is not known whether the cytotoxic effects of tTG occur exclusively in the cytosol, at cellular membranes, or both. However, a set of studies by Hwang and coworkers suggests a simpler possibility, namely that tTG might not be in the closed-state when participating in GPCR signaling pathways.

**Fig. 5:**
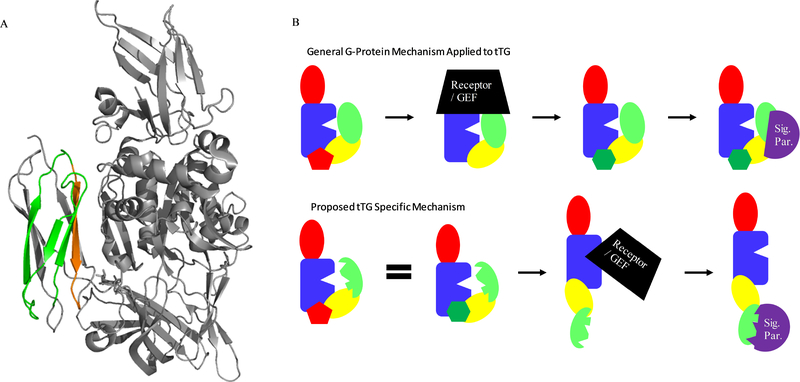
tTG behaves in some ways like a classical G-protein. (A) A peptide matching the orange sequence at the C-terminus of tTG is able to block binding between tTG and PLC, which it binds following activation by the α1-adrenergic receptor. Peptides matching regions shown in green do not block the interaction. The sequence in orange is inaccessible to solvent, and presumably binding partners, while tTG is in the closed-state. (B) Proposed mechanism by which tTG signals. For a classical G-protein, such as Gαq, the protein normally exists in a GDP (red pentagon) bound state. Receptors bind and stimulate GDP dissociation. The protein then rapidly binds GTP (green hexagon), which is in excess relative to GDP in cells. This causes a structural change (which is not seen in tTG crystal structures), and allows binding and activation of downstream signaling partners (“Sig. Par.”, shown in purple). We suggest a possible alternate mechanism in which the nucleotide bound forms of tTG are all structurally similar, and have effectively identical signaling ability. Receptors which bind to the open-state of tTG cause the dissociation of both GDP and GTP, and reveal the inner-face of the C-terminus of the protein to solvent, which then specifically binds downstream signaling partners.

Specifically, Hwang *et al.* found that phospholipase C (PLC) co-immunoprecipitated with wild-type tTG, and that a peptide matching residues 661–672 of tTG could prevent this interaction, while peptides containing residues between 618 and 661 were unable to prevent binding ^[83]^. As shown in Fig. 5A, the inhibitory peptide matched a region of tTG inaccessible to solvent in the closed-state, while the ineffective peptides would be accessible when tTG is bound to nucleotide. A more recent study by Feng *et al.* showed that this peptide, attached to a resin, can successfully precipitate PLC ^[84]^. Given these findings, and the previously described studies, it seems possible that receptors which bind tTG (*i.e.*, Gα_h_) effectively accomplish only the first step of a normal nucleotide exchange reaction, *i.e.*, catalyzing the loss of GDP from tTG. However, instead of GTP then binding to the protein, it remains in a nucleotidedepleted state and adopts the open-state, allowing access to binding partners. This proposed process is shown in Fig. 5B.

## 7 CONFORMATION-INDEPENDENT BINDING MODES OF TTG

**Fig. 6:**
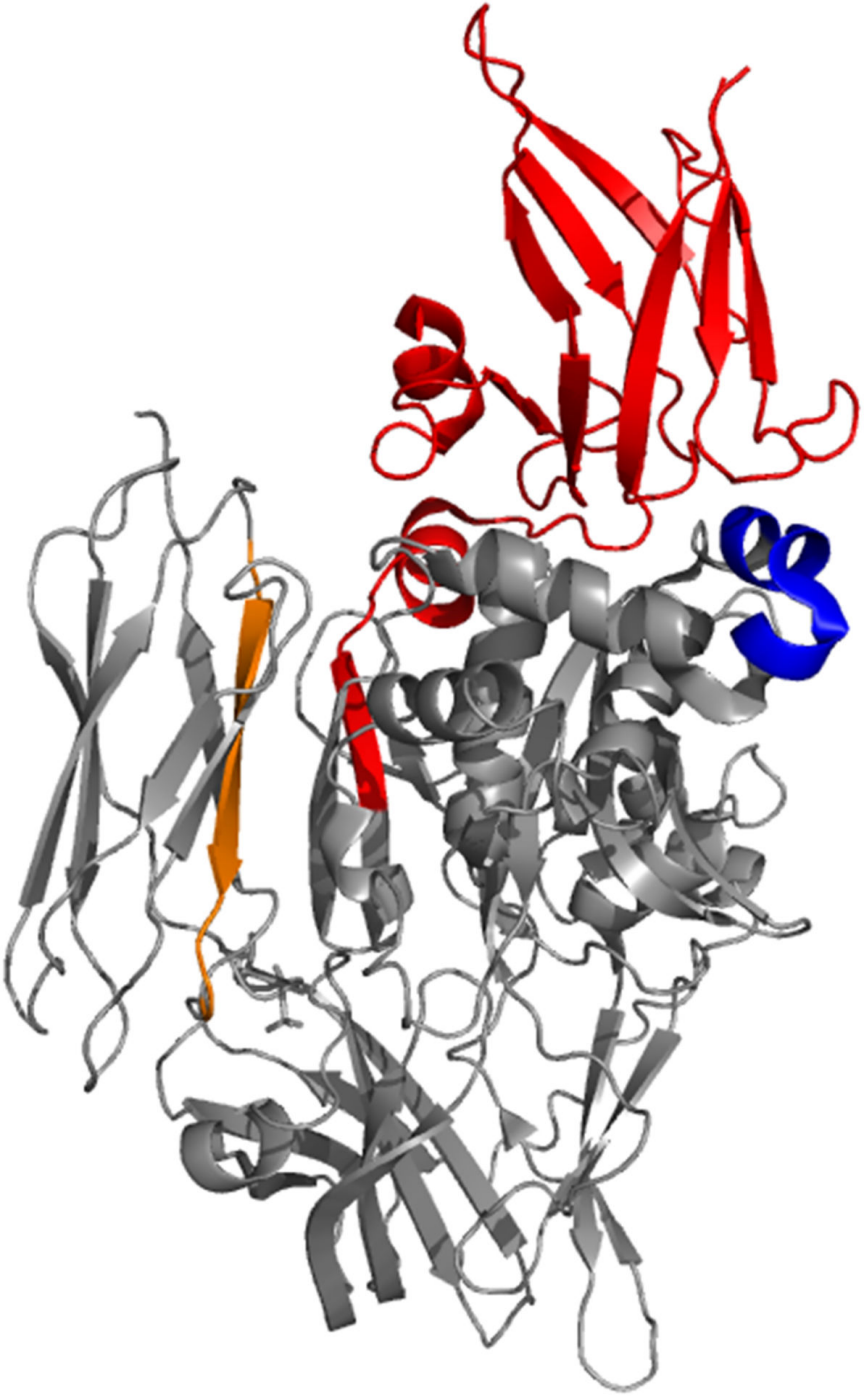
Regions of tTG associated with binding partners. Fibronectin binds to tTG via it’s N-terminal region (colored red), while the blue colored region is a BH3 domain, and binds proteins such as Bax. In orange is the region also shown in Fig. 5, from which a peptidomimetic can be made that binds PLC.

Other aspects of tTG function may be independent of its conformation, and are thus presumably not the source of tTG open-state cytotoxicity. For example, in 2011, Boroughs and coworkers reported that tTG localized to the leading edges of HeLa cervical carcinoma cells in an HSP70-dependent manner to promote cell migration. This function, however, occurred with wild type tTG, tTG C277V, and tTG R580K ^[85]^, and was presumably independent of tTG conformation. Similarly, in 1995, Jeong *et al.* demonstrated that tTG bound to fibronectin via its N-terminal 28-kDa fragment, as isolated following proteolysis by the endoproteinase Glu-C (Fig. 6) ^[86]^. Akimov and coworkers later expanded upon this result by showing that a recombinantly expressed tTG fragment, residues 1–167, was sufficient to bind fibronectin ^[60]^. These residues are exposed regardless of tTG conformation, and so it is expected fibronectin would bind tTG in either the open- or closed-state.

In some other cases, a binding interaction occurs with tTG in either conformational state, but has different effects based upon its catalytic activity. One such case involves its BH3 domain. In 2004, Rodolfo and colleagues demonstrated that tTG had a functional BH3 domain along the outer face of the crosslinking-catalytic domain (Fig. 6) ^[87]^. The authors demonstrated that tTG was able to interact with the pro-apoptotic protein Bax via co-immunoprecipitation experiments. They also showed that expression of tTG sensitized SK-n-BE cells to staurosporine induced cell death, but that cells expressing tTG lacking the BH3 domain, or with a mutated BH3 domain, were not sensitized. Further, cells expressing tTG C277S were not sensitized, showing that tTG crosslinking activity was necessary to enhance the cell death response, and Bax was shown to be a crosslinking substrate for tTG.

Indeed tTG has long been noted to have roles in both promoting cell survival and driving apoptosis ^[2,25,88]^. Since cells are flooded with calcium from the endoplasmic reticulum and mitochondria as part of apoptosis ^[89,90]^, and because calcium levels are relatively low in healthy cells ^[39,40]^, it is reasonable to expect tTG to be predominantly in the closed-state in healthy cells (*i.e.,* when it is promoting cell survival) and in the open-state when it is helping to drive cell death. In the case of Bax, it is probable that after binding to Bax via its BH3 domain, closed-state tTG sequesters it, similar to c-Cbl ^[67]^, while open-state tTG crosslinks Bax into large aggregates which form pores in the outer mitochondrial membrane. Yoo and coworkers have also demonstrated that tTG is necessary for the localization of Bax to the mitochondrial membrane ^[91]^. However, a conflicting report by Cho *et al.* showed that tTG actually downregulated Bax in HEK293 cells following treatment with A23187, an ionophore that floods cells with calcium to induce cell death ^[92]^. Similarly, tTG has been shown to promote cell survival by crosslinking p110 Rb, which it could only do in the open-state ^[65,66]^. Thus, there must exist at least one regulating factor that shifts open-state tTG from a pro-survival to a prodeath protein. It is currently unclear if constitutively open-state tTG bypasses such a factor.

## 8 A ROLE FOE TTG SHORT?

A number of groups primarily interested in diseases of the central nervous system have identified a tTG splice variant which is now known as tTG-short, or tTG-S, and may provide some clues as to why open-state tTG is cytotoxic. In 1992, Fraij and coworkers isolated the cDNA for a short isozyme of tTG from human erythroleukemia cells ^[93]^. This shorter variant was identical to tTG, except that it lacked the C-terminal 139 residues. Monsonego *et al.* demonstrated the existence of a similar shorter splice variant of rat tTG isolated from astrocytes, and although this variant only lacked 34 C-terminal residues relative to the longer canonical sequence, it was suggested that removal of these residues might be a common splice-variant form for tTG across mammalian species ^[94]^. This study also showed that GTP binds more weakly to the shorter tTG-S variants compared to the longer tTG. This inability to bind nucleotide strongly suggested that tTG-S adopted a conformation similar to the open-state. Indeed, Singh *et al.* found that the SAXS envelope of tTG-S fit a dimer of two open-state proteins ^[74]^.

Citron, Festoff, and coworkers published a series of studies in which they found that tTG-S was expressed in the brain of Alzheimer’s disease patients ^[95–97]^. They latter reported that tTG-S expression was rapidly induced following spinal cord injury in rats, peaking within 24–72 h of injury ^[97]^. Both Alzheimer’s disease and spinal cord injury tend to lead to apoptosis in affected cells, and the authors found that tTG-S expression occurred before the onset of apoptosis in spinal cord injury. The authors noted that tTG has been frequently observed to be upregulated during apoptosis, which causes increases in intracellular Ca^2+^ levels, thereby activating tTG, which in turn crosslinks proteins to form apoptotic bodies. It was further suggested that nucleotide-binding-deficient tTG-S was expressed to help begin the process of apoptosis before sufficient Ca^2+^ had flooded the cell to activate normally present tTG. tTG-S has been shown to have very little native crosslinking ability compared to wild-type tTG ^[98]^, and so if this hypothesis is true, it would most likely be due to a direct binding interaction, and not tTG crosslinking activity.

Tee *et al.* in 2009 demonstrated that human neuroblastoma cells expressed both tTG and tTG-S, which showed opposite effects on cell differentiation, *i.e.*, tTG inhibited differentiation while tTG-S enhanced it ^[99]^. Cell differentiation was also promoted by tTG R580A, suggesting that either the conformational state of tTG or its crosslinking activity was responsible for enhancing this cellular outcome. The authors thus conducted experiments with cystamine, an alternative tTG substrate which inhibits its on-target crosslinking activity, and demonstrated that treating cells with cystamine prevented the effects of tTG R580A and tTG-S, suggesting that they enhanced differentiation primarily via their crosslinking ability. This matched data from Tucholski and coworkers, who had demonstrated that SH-SY5Y neuroblastoma cells overexpressing tTG were able to differentiate, but differentiation was prevented by overexpressing the crosslinking-defective tTG C277S or by inhibiting tTG biosynthesis with shRNA ^[100]^. However, in 2006, Antonyak *et al.* reported that ectopic expression of tTG-S in NIH-3T3 cells was highly cytotoxic, and that these effects remained when tTG-S C277A was transiently transfected into the cells ^[98]^. Having demonstrated that the crosslinking activity of tTG-S was not responsible for its ability to kill cells, it was then demonstrated that tTG-S formed large aggregates in cells, suggesting that this aggregation might be responsible for the cell death. Moreover, a more recent report from Fraij demonstrated that transfection of an even shorter tTG variant, containing residues 1–464, into MCF7 or T47D breast cancer cells greatly increased the degree of apoptosis when the cells were cultured in serum-free medium ^[101]^. Further, Fraij demonstrated that addition of cystamine partially blocked the apoptosis-enhancing effects of the shortened tTG. It is currently unclear if these different biological outcomes are due to small differences in the techniques used in these studies, or reflect the unique roles tTG might play in astrocytes, neurons, and other tissues ^[41]^. What seems clear, however, is that tTG-S resembles the open-state tTG, and it promotes cell death in many scenarios.

## 9 EXPLOITING THE OPEN-STATE OF TTG

In some cases, such as Alzheimer’s or celiac disease, the open-state of tTG appears to be responsible for promoting the disorder. In Alzheimer’s disease, tTG can crosslink Abeta, which leads to dangerous plaques ^[45,47]^, while in the case of celiac disease tTG transforms gluten peptides into immunoreactive species ^[4,102,103]^. However, targeting the open-state of tTG could be of particular therapeutic value in cancer. tTG is overexpressed in many of the most aggressive cancers ^[2]^, and tTG knockout mice are predominantly healthy, with their phenotypes being variously reported as normal ^[104]^, or only slightly disrupted ^[105]^, and so tTG inhibitors would be expected to be minimally toxic. Moreover, the cytotoxicity of open-state tTG suggests that pharmacological stabilization of the tTG open-state in tumors could be effective against rapidly growing, deadly cancers such as those of the brain, pancreas, and lung.

Thus far, this hypothesis has not been tested thoroughly. The cytotoxic open-state of tTG is still poorly understood, and so efforts to intentionally induce this state in cells have been minimal. However, a number of small molecules have been reported which stabilize the open-state of tTG. Chief among these are peptidomimetic inhibitors. Because of the role tTG plays in celiac disease ^[4,7,106]^, and its related modification of gluten peptide, a great deal of effort has gone into the design of peptidomimetic inhibitors of tTG ^[2,23,28]^. Peptidomimetic compounds typically have a number of strengths (tight binding, high specificity, ease of synthesis), as well as weaknesses (high molecular weight, low cell permeability, metabolic instability), and those developed for tTG tend to exhibit both. Still, they have one advantage that peptidomimetic compounds targeting other proteins do not share: they induce the open-state of tTG.

**Fig. 7:**
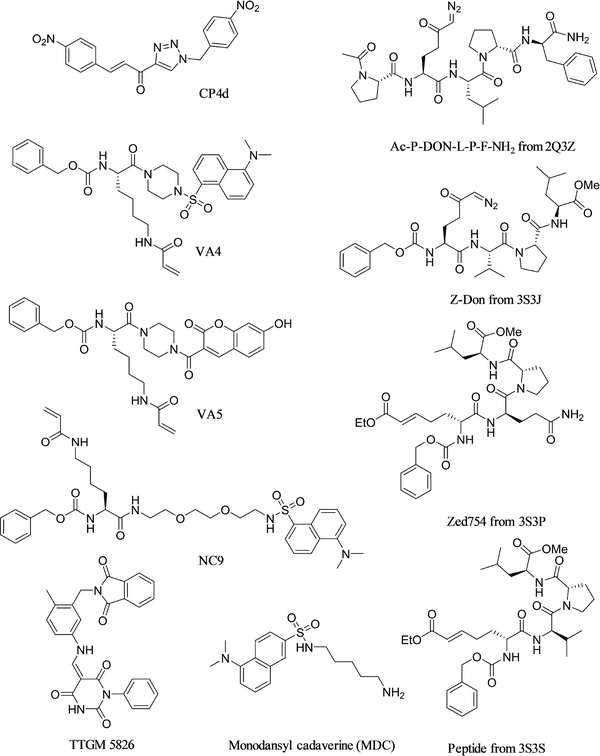
Inhibitors of tTG stabilize the protein in the open-state. Ac-P-DON-L-P-F-NH2, Z-Don, Zed754, and a peptidomimetic similar to Zed754 (right side) were all demonstrated to stabilize the open-state of tTG via X-Ray crystallography ^[35,107,108].^ VA4, VA5, NC9 ^[109],^ MDC ^[67]^, and TTGM 5826 ^[110]^ were each demonstrated to stabilize the open-state of tTG through assorted biochemical assays. Notably, very large amounts of MDC were needed to stabilize the tTG open-state. CP4d has been reported to modestly stabilize the closed-state of tTG ^[73]^ or the open-state of tTG ^[109]^ depending upon the experimental system, and its true effects on tTG structure are at best inconclusive currently.

The first crystal structure of tTG in the open-state (PDB code 2Q3Z) included an irreversible peptidomimetic inhibitor, “Ac-P-DON-L-P-F-NH_2_” (Fig. 7) ^[35]^, based on the sequence “P-Q-L-P-Y”, which is found multiple times in gluten proteins. Since then, three more crystal structures have been reported in which tTG is bound to a peptidomimetic inhibitor: 3S3J, in which tTG is bound to the compound Z-Don ^[107]^, 3S3P, in which tTG is bound to the molecule ZED754 ^[108]^, and 3S3S, in which tTG is bound to a similar irreversible peptidic compound (Fig. 7). In all four cases, tTG was crystallized in the open-state, but no calcium ions were found in the crystal structure, strongly suggesting that any peptidomimetic compound of similar size (in these cases, ~5 residues) would stabilize the open-state.

Further evidence that irreversible peptidomimetic compounds stabilize the open-state of tTG comes from a recent report by Kerr and coworkers ^[109]^. Kerr examined four compounds: the irreversible peptidomimetics NC9, VA4, and VA5, and the reversible small molecule CP4d (Fig. 7). Using the mCer-tTG-YFP construct discussed above, the authors were able to demonstrate that all three of the peptidomimetic compounds stabilized cellular tTG in the open-state conformation. They demonstrated similar results when using GTP-agarose beads to pull down closed-state tTG, in which case they saw less protein bound to the beads when treated with the three inhibitors. Similarly, application of the inhibitors resulted in a decrease in closed-state tTG as measured by native state electrophoresis.

Unlike the three peptidomimetic inhibitors studied by Kerr, the effect of CP4d on tTG conformation is less clear ^[109]^. In earlier studies, Caron *et al.* had shown that CP4d was able to stabilize the closed-state of tTG according to FLIM-FRET measurements using the mCer-tTG-YFP construct ^[73]^. Kerr’s study, however, found no statistical significance when these measurements were repeated. Measuring the conformation of recombinantly expressed tTG by shifts in gel electrophoretic mobility showed that CP4d was able to stabilize the open-state of tTG, but to a lesser extent than the peptidomimetics, and the authors considered the molecule’s impact on tTG conformation to be minimal ^[109]^. Other non-peptidic molecules have been shown to have a greater ability to stabilize the open-state of tTG, however. Zhang *et al.* measured the effects of the alternate substrate MDC on tTG conformation ^[67]^. Based on the proteolytic degradation rate of recombinant tTG, they determined that MDC was able to stabilize the open-state of the enzyme, at concentrations of 0.5–1 mM. More recently, the small molecule TTGM 5826 was reported (Fig. 7) ^[110]^. This reversible, non-peptidic molecule was discovered via virtual screening efforts against the crystal structure of open-state tTG in an effort to find a molecule that would stabilize that conformation of the protein. It was shown to stabilize the open-state of tTG by both nucleotide binding and proteolytic degradation assays. Like the peptidomimetic inhibitors, it inhibited the growth of a variety of cancer cells, with a similar potency as that giving rise to a stabilization of the tTG open-state (an IC_50_ value of approximately 20–30 μM). While this small molecule has not yet been as robustly investigated as peptidomimetic compounds such as NC9 or Z-Don, it provides a proof-of-concept that reversible small molecules can stabilize the cytotoxic open-state of tTG, and hopefully will spur new developments in that arena.

## 10 OPEN QUESTIONS

We have tried to highlight several areas deserving of more research while examining the studies to date pertaining to the open-state of tTG. In fact, a number of wide-ranging questions remain. Two come from the Begg work in 2006 ^[40]^, which involves two interesting, although counter-intuitive, pieces of data. The first involves their ITC experiments with tTG and GTP-γS. The authors demonstrated that the binding stoichiometry between nucleotide and tTG was roughly 1:3. This leads to the question of whether tTG forms a higher order structure, with one closed-state molecule somehow causing two other tTG molecules to adopt the closed-state, while simultaneously blocking access to their nucleotide binding pockets. Such a polymer might be similar to those observed with open-state tTG by Kim and colleagues ^[77,111]^, and could have important consequences for tTG binding interactions in cells. A second question pertains to constitutively open tTG mutants such as rat tTG R579A (equivalent to R580A in humans). Experiments aimed at determining transamidation activity as a function of Ca^2+^ concentration showed that the EC_50_ for calcium activation was essentially invariant with respect to tTG conformation ^[40]^. Specifically, the calcium EC_50_ for wild type tTG was 507 μM, while that for tTG R579A was 478 μM. This then raises questions as to whether open-state tTG mutants have crosslinking activity in most cancer cells that requires calcium to be activated, and what additional functions might calcium play in tTG activation beyond stabilizing the open-state and stimulating transamidation activity.

**Fig. 8:**
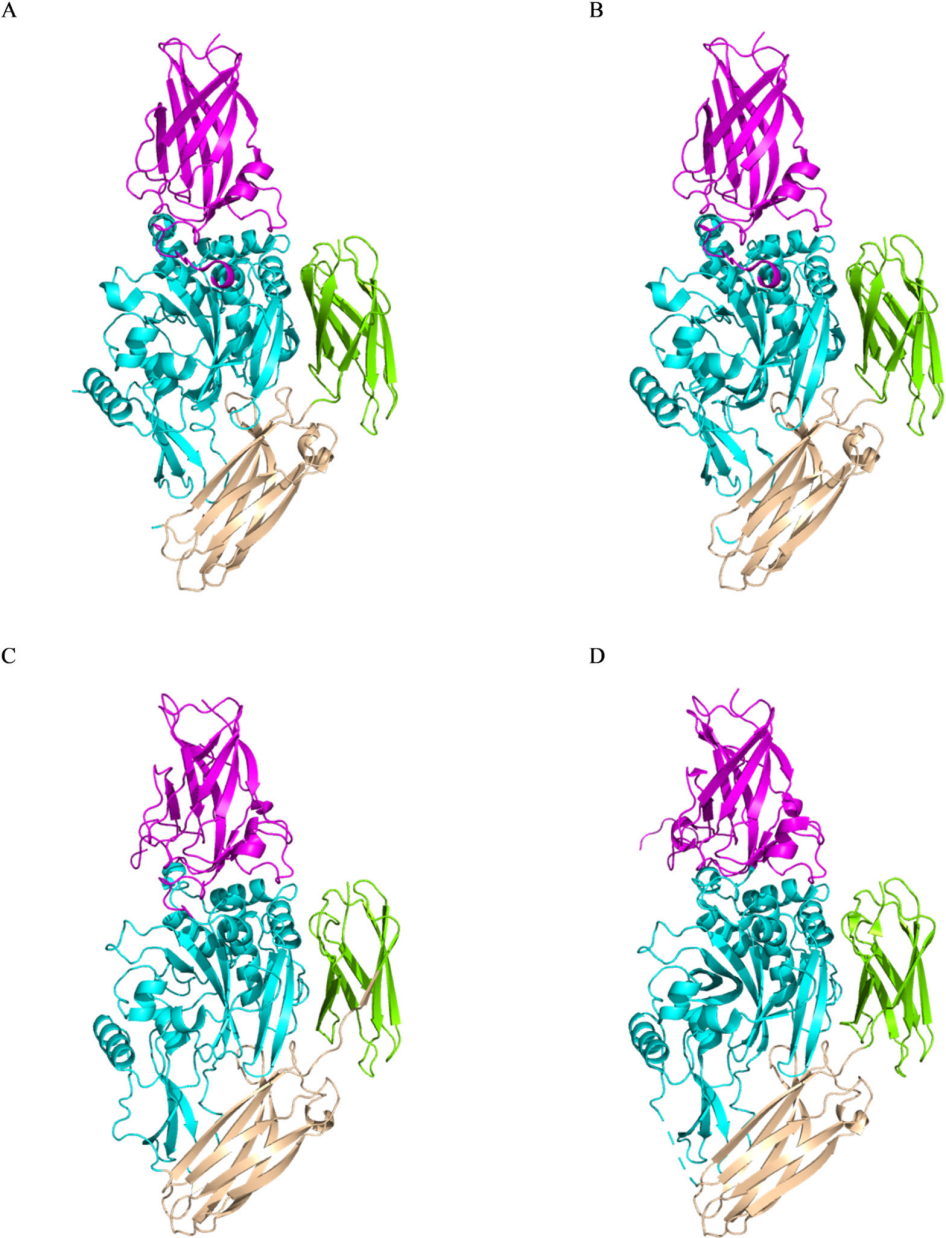
X-Ray crystal structures of close homologues to tTG. X-Ray crystal structures have been solved of (A) inactive TG3 (PDB code 1NUG), (B) active TG3 (PDB code 1NUD), (C) inactive Factor XIIIA (PDB code 1FIE), and (D) active Factor XIIIA (PDB code 1EVU). Each protein has four domains, substantially similar to tTG, but neither has the radical conformational shift of tTG upon binding calcium to adopt an activated state.

Another major question pertains to other members of the transglutaminase family. There is substantial homology between the different transglutaminases ^[2]^. Further, other transglutaminases have been crystallized, and found to have secondary structures similar to that of tTG. Consider, for example, Factor XIII-A (Fig. 8A,B) ^[112,113]^, and TG3 (Fig. 8C,D) ^[75,76]^. Each protein has a structure very similar to that of tTG, and is activated by calcium. However, crystal structures of each enzyme have been solved with and without calcium bound, with all of these structures appearing to be very similar (Fig. 8). Do any other transglutaminases have the dynamic conformational changes exhibited by tTG? If so, do their open-state conformations cause a cytotoxic effect in cells?

Still another question concerns how to exploit the open-state of tTG in cancer, or protect against it in neurodegenerative diseases. In cancer, inducing the cytotoxic open-state of tTG would be highly desirable. With the single exception of TTGM 5826, the only inhibitors which have so-far been shown to do this potently are irreversible peptidomimetics. Peptides largely tend to have poor cell permeability, while irreversible inhibitors require a significant time period to be eliminated from the patient, increasing the risk of toxic effects. This would be particularly important in the case of tTG, because a cytotoxic effect is being induced. Similarly, since the mechanism of open-state cytotoxicity is so poorly understood, a large peptidic inhibitor might block critical binding interactions. As such, it would be desirable to develop alternate scaffolds to stabilize the open-state of tTG. In diseases such as Alzheimer’s or Parkinson’s, however, the open-state of tTG has deleterious effects that might cause unintended and undesirable cell death throughout the brain. Inhibitors which stabilize the closed-state of tTG, perhaps by tightly binding to the nucleotide pocket, could be of tremendous value in that field.

Finally, and perhaps most importantly, is the question “why is open-state tTG cytotoxic?” What are the key binding partners of open-state tTG, versus closed-state tTG, and how do these different partners lead to such markedly different biological outcomes? We have discussed several features of open-state tTG which may account for some of its cytotoxicity, but none seem to be the unambiguous and definitive cause of its toxic effects. Given the prevalence of tTG in so many diseases, and its presence in so many tissues, answering these questions could be extremely valuable and give rise to new therapeutic strategies.

## Figures and Tables

**Table 1.** Conformation adopted by several mutants of tTG. The indicated mutants of tTG (arranged by residue number) are thought to stably adopt the open-state or closed-state conformation, or to have unchanged conformational stability relative to wild type tTG, as listed. Note that the conformation of each mutant is primarily deduced from measurements of GTP-binding capability, crosslinking activity, and proteolytic stability (not tabulated), and with rare exception has not been unambiguously determined via direct structural study.

Mutant	Probable conformation	GTP-binding?	Transamidation activty?	Reference
Q163L	Partial Open	Partial −	Partial +	[71]
Q163D	Partial Open	Partial −	Partial +	[71]

Q164L	Partial Open	Partial −	Partial +	[71]
Q169 L	Partial Open	Partial −	Partial +	[71]
S171A	No Change	+		[40]
S171C	No Change	+	+	[71]
S171E	Open	−	+	[71,72]
K173L/R/N	No Change	Slight −	+	[70]
K173 L/N	Open	−	+	[71]
F174A	Open	−	+	[40]
F174W	No Change	+		[40]
W254A	Open	–		[74]
C277V	Open	−	−	[67]
C277A	Open	−	−	[72]
C277S	No Change	+	−	[39]
D306N, N310A	Closed	+	−	[8,67]
R476A	No Change	+	+	[40]
R478L	Open	−	+	[70]
R478A	Partial Open	Partial −	+	[40,72]
Y516F	Partial Open	Partial −	+	[72]
R579A (rat)	Open	−	+	[40]
R579K (RAT)	Open	−		[40]
R580K	Open	−	+	[67,70]
R580L	Open	−	+	[70]
R580A	Open	−	+	[39]
N681A	Open	−		[74]
